# Pulsed artificial light at night alters moth flight behaviour

**DOI:** 10.1098/rsbl.2024.0403

**Published:** 2024-11-13

**Authors:** Madeleine Fabusova, Kevin J. Gaston, Jolyon Troscianko

**Affiliations:** ^1^ Centre for Ecology & Conservation, University of Exeter, Penryn Campus, Penryn, TR10 9FE, UK; ^2^ Environment and Sustainability Institute, University of Exeter, Penryn Campus, Penryn TR10 9FE, UK

**Keywords:** artificial light at night, pulsed light, moths, flight behaviour, headlights

## Abstract

Vehicle headlights create pulsed artificial light at night (pALAN) that is unpredictable, intense and extends into previously dark areas. Nocturnal insects often have remarkable low-light vision, but their slow pupillary light responses may leave them vulnerable to pALAN, which has important ecological consequences. To test this, we exposed nocturnal moths—important pollinators and prey—to four pALAN treatments. These comprised ‘cool’ and ‘warm’ lights, either emitted from phosphor-coated light-emitting diodes (LEDs) or RGB (red-green-blue) LEDs, matched in colour (CCT) and intensity to human vision. We assessed the initial behavioural response, likely crucial to the survival of an organism, of 428 wild-caught moths comprising 64 species. We found that exposure to a cool phosphor-coated LED light pulse increased instances of erratic flight and flight-to-light that are likely detrimental as they increase the risks of impact with a vehicle, predation or excess energy expenditure. Our findings suggest that pALAN can cause a wide range of behavioural responses in nocturnal moths, but that the most harmful effects could be minimized by reversing the current shift towards high CCT (cool) phosphor-coated LED car headlights. Lower CCT or RGB alternatives are likely to provide benefits for road safety while reducing ecological harm.

## 1. Introduction

Natural light regimes are being disrupted globally due to the introduction of artificial light at night (ALAN) [1,2], raising the intensity of night-time lighting while altering its spatial and temporal properties [3]. Given the key roles that natural light regimes play as a resource and source of information [4], the biological impacts of ALAN would be predicted to be marked, particularly in taxa such as insects that are largely nocturnal [5]. Indeed, the impacts of ALAN have been found at the individual (e.g. physiology and behaviour), population (e.g. abundance, distribution), community (e.g. species richness and composition) and ecosystem (e.g. function and services) levels, and across a very broad range of higher taxa [1–12]. Despite the array of research on the impacts of ALAN, to date, this has almost exclusively focused on static sources of light emissions, such as streetlights (or their proxies), or experimental introduction of light to previously dark field sites [12–14]. However, ALAN is also produced from moving light sources, in particular, vehicle headlights that form horizontally focused beams that are typically an order of magnitude more intense than static streetlights [6,15,16]. Organisms will tend to experience this as pulsed light (hereafter pALAN), of varying frequency and intensity dependent on traffic levels, as these sources pass by [16]. The expansive nature of road networks, combined with the potential for focused headlight emissions to propagate over long distances means pALAN can occur over vast areas, including rural settings of conservation concern. By one estimate, 70% or more of the land of Great Britain might be affected to some degree [17].

Following exposure to a pulse of light, vertebrate pupils constrict to reduce the aperture [18], a process that takes roughly 1 s in humans. This pupillary light reflex (PLR) is affected by the interplay between the levels of darkness before light exposure, the extent of the retina stimulated, the colour of the light (and interactions with ambient light levels) and the age of the individual [19,20], leading to decreased alertness in older people when exposed to ‘cool’ blue lights [21]. The PLR mechanisms found in nocturnal insects are markedly different. Their vision adapts by migrating screening pigment granules in the optical units radially (in apposition eyes) or longitudinally (in superposition eyes), which can take over 30 min to return to a dark-adapted state after light exposure [22]; insect PLR mechanisms have also been found to operate more slowly at night than in the daytime [23]. The spectral response of the PLR has been found to vary among closely related hawkmoth species and even among individuals of the same species [22,24]. The speed of these pupillary movements will determine the insect’s ability to respond [25], and these initial responses following a pALAN encounter are likely to affect survival (e.g. by inhibiting natural escape, mating or pollination behaviour). Taken together, this could make nocturnal insects vulnerable to pALAN from car headlights, particularly given recent trends towards more intense, blue-shifted headlights that use phosphor-coated LED technology with a peak in the shortwave range [16,18,19,21–26].

Here, we assessed the initial behavioural responses of nocturnal moths to four experimental pALAN treatments, comprising two ‘correlated colour temperatures’ (CCT; ‘cool’ and ‘warm’), and two light-emitting diode (LED) spectral emission shapes (phosphor-coated and RGB), together with a dark control. A number of different mechanisms could govern these responses: First, the ‘blinded-by-light’ hypothesis predicts that a combination of slow PLR and photobleaching (the depletion of photopigment by exposure to bright light [27]) causes the loss of visual flight stabilization feedback, leading to erratic uncontrolled flight, most likely towards the light source as it is the only visible landmark. Second, the ‘positive phototaxis’ hypothesis predicts that the flight control system causes flight towards the light, with the moth orienting dorsally towards the light source [28], subsequently causing blinding and erratic behaviour (as above) or resting on or near the light. Third, the ‘negative phototaxis and/or activity suppression’ hypothesis predicts that the light triggers a ‘daytime’ response, causing the moth to seek shelter or stay put (high latency to move; a light above a certain brightness can elicit daytime light adaptation [29,30], suppressing activity [31,32]). Finally, a ‘predation escape response’ may be elicited due to the handling of the moths that could manifest as shelter-seeking or flight upwards/away. Such a response could supersede others, or cause interactions. Each of these responses is potentially harmful to the moths; however, those that cause erratic flight or draw individuals towards the light source are likely to be most harmful, increasing the risk of impact with a vehicle, predation or excess energy expenditure.

## 2. Methods

### (a) Data collection

**Figure 1.**
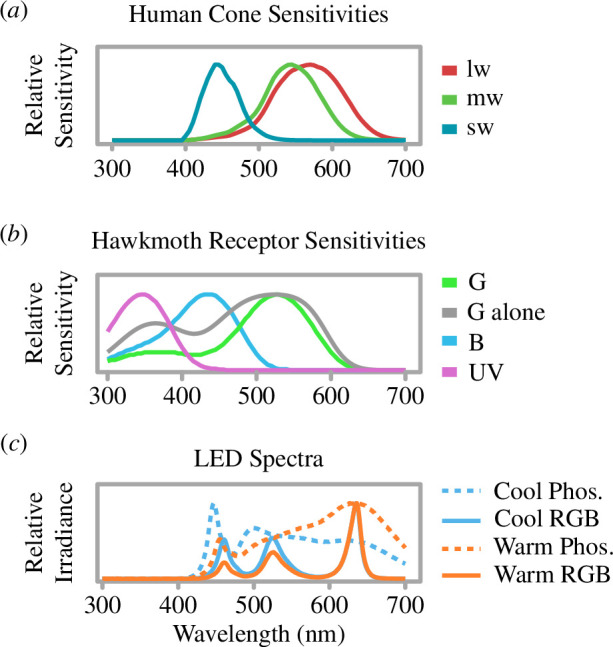
Spectral sensitivity of (*a*) cones in humans [33,34]*,* with long- (lw), middle- (mw) and short-wavelength-sensitive cones (sw), referred to based on the peak sensitivity, presented; and (*b*) photoreceptors of the nocturnal elephant hawkmoth (*Deilephila elpenor*) [35,36], with ultraviolet (UV), blue (B) and green photoreceptor (G) sensitivities shown. Additionally, green photoreceptor sensitivity typically used for achromatic contrast calculations is depicted by the ‘G alone’ line; and (*c*) irradiance plots of experimental treatments. Graph (*c*) was rescaled to max = 1.

Experiments were conducted over four months (June–September 2023) at night at Tremough Campus, Penryn, Cornwall, UK. Each trial consisted of a single moth being released and simultaneously exposed to a 10 s pulse of light with one of four experimental treatments, selected randomly but balanced per treatment. A subset of experiments also used a no-light control (not balanced per treatment but controlled for statistically). The experimental treatments were: cool phosphor-coated LED (5664 K; 454.9 lx), warm phosphor-coated LED (3102 K; 429.7 lx), cool RGB LED (5661 K; 433.5 lx) and warm RGB LED (3222 K; 463 lx) (Neewer 2 RGB)—i.e. two different light CCTs achieved with two different emission spectra. Irradiance was measured using a Jeti Specbos 1211-UV spectroradiometer, and the small discrepancies in brightness between treatments were further controlled by adjusting the distance between the light and moth release point. The light colour temperature (CCT) is measured in kelvin (K), and high values indicate cooler (shortwave-shifted) lights. CCTs were matched to human vision between the ‘warm’ and ‘cool’ treatments by finding metameric colours (where two different spectra are perceived as identical colours to a given viewer). This analysis uses human visual metrics because sources of ALAN are ultimately designed for human visual performance (see electronic supplementary material, T1). The irradiance of treatments is compared with the visual sensitivities of a human and a nocturnal hawkmoth (elephant hawkmoth, *Deilephila elpenor*) (figure 1). It is important to note that the light source was static, but the short pulses aimed to simulate passing vehicle headlights and moths encountering these at close proximity.

Moths used in the experiments were wild-caught using a red LED headtorch (Lighting EVER 3200008-2). Colour vision has been well described in hawkmoths, which show low sensitivity to red wavelengths [37] (figure 1); colour vision in other moth taxa is poorly understood, and we cannot rule out red light sensitivity. Moths were caught at night with a butterfly net to avoid using more conventional light traps that can affect moth behaviour and lead to biased sampling of insect assemblages [38]. Five pre-determined transects were sampled in random order for 10 min, starting half an hour after sunset (for a map of transects, see electronic supplementary material, S1). Ambient light varied from directly beneath streetlights, to darker areas of campus (where sky-glow is rarely lower than 0.05 lx on overcast nights). Moths were immediately identified to the species-level using [39,40], or where this was not possible, photographs were taken in ambient low-light for later identification. Caught moths were placed in an insect-collecting pot with no lighting for at least 10 min to dark-adapt them before experiments.

Environmental data, including temperature, humidity and wind speed (m s^−1^), were obtained from a weather station at the study site (on the Environment and Sustainability Institute), with measurements averaged over the recording period (approximately 2 h each data collection day). Moon phase was obtained from https://www.timeanddate.com/moon/phases/.

### (b) Experiments

At the start of each experiment, each pot was opened and directed at the light, and the moth was exposed to 10 s of light (no light for control), from a distance of 1 m or 1.06 m, respectively (see electronic supplementary material, S1). Moths were tested in the same area and under the same ambient light levels where they were caught in flight. Latency was measured as the delay (in seconds) between the end of the exposure to the light (or 10 s control) and each behavioural response. Observations were made by M.F. using a red light headtorch and a voice recorder for later transcription. Each moth was assigned an initial behavioural response based on pilot studies (*n* = 72) that identified the five most common such responses: *erratic flight—*fast zig-zag response with no particular direction; *flight-to-light—*flight into the light source, associated with hitting it or perching on it; *positive geotaxis*—downward flight, associated with seeking shelter in low vegetation; *negative geotaxis—*upward flight, associated with immediate return to pre-light exposure flight activity; and *no response—*indicated by staying in the pot for the duration of the experiment. Trials ended after 2 min, and moths that elicited no response were released.

### (c) Data analysis

Linear and generalized linear mixed-effects models with binomial error structures were used to analyse the effects of experimental treatments on initial behavioural responses, using species and family as random factors to control for relatedness. Models were checked for overdispersion. Results from controls were used to demonstrate an overall effect of pALAN on moth behaviour compared with darkness; however, to investigate the effects of CCT and spectrum independently, and their interactions, we removed control data for subsequent analyses. Seasonality was controlled for by including environmental variables (temperature, humidity, wind speed and moon phase) as additive fixed effects in the GLMMs. Full interactions between experimental treatment levels and the behavioural response (response~LED type*CCT) were included in the models, and these were simplified using the F-test or chi-square (*X*^2^) to create minimal adequate models. Chi-square statistics all have d.f. = 1 (model comparisons) unless otherwise stated. Significance was set to *p* < 0.05. Data were analysed to compare levels of initial response: erratic flight (binary response = 1) versus other behaviour (0) (*n* = 386); erratic flight and flight-to-light (1) versus other behaviour (0) (*n* = 386); negative geotaxis (1) versus positive geotaxis (0) (*n* = 199, other behaviours excluded). The latency to respond was log-transformed for analyses. All analyses were also carried out separately with the four most abundant moth families (see electronic supplementary material, T2, figures S2 and S3).

Statistical analysis and visual modelling were carried out in R version 4.3.1 [41] and included the following packages: tidyverse 2.0.0 [42], pavo 2.9.0 [43], nnet 7.3.19 [44], nlme 3.1.162 [45], lme4 1.1.35.1 [46] (for full statistical model outputs and visualization packages, see electronic supplementary material, T3).

## 3. Results

A total of 428 individual moths were tested (cool phosphor-coated LED *n* = 100; warm phosphor-coated LED *n* = 92; cool RGB LED *n* = 98; warm RGB LED *n* = 96; control *n* = 42). These represented 64 species from seven families (electronic supplementary material, T2; Crambidae *n* = 48, Erebidae *n* = 147, Geometridae *n* = 126, Noctuidae *n* = 72, Tortricidae *n* = 24, Pyralidae *n* = 10, Drepanidae *n* = 1). Temperature and humidity ranges were small but suitable for activity [47], and these were the only environmental predictors of response and thus kept in the models. The detailed statistical outputs are provided in the electronic supplementary material, tables S4–S19.

**Figure 2.**
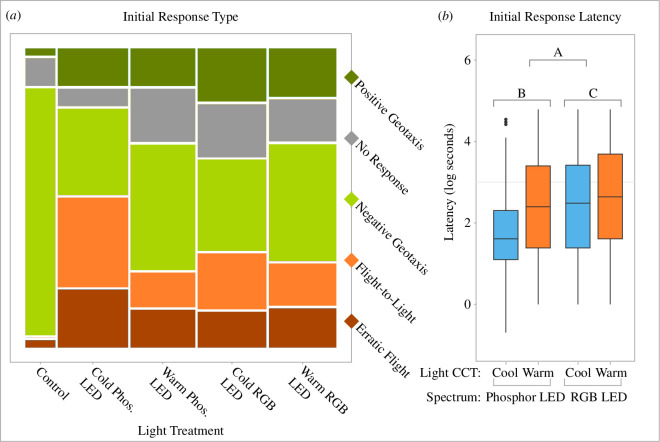
(*a*) Frequency of initial behaviours under all pALAN treatments and control, displayed in a mosaic plot, where the width of the column indicates the number of repeats. (*b*) Latency (log-transformed) of moths’ responses under the different treatments. The lines in the box plots represent the medians; the box represents the interquartile range (IQR), the upper (Q3) and lower (Q1) quartiles; the whiskers show the range of the data (minimum and maximum value excluding outliers, respectively, with dots showing outliers). The letters above the plots indicate the following: A indicates the spectrum differences between phosphor-coated LEDs and RGB LEDs, with *p* = 0.003; B indicates the statistical comparison (Bonferroni *post-hoc* test) between cool and warm phosphor-coated LEDs, with *p* = 0.001, and C indicates the statistical comparison between cool and warm RGB LEDs, with *p* = 0.392.

Moths demonstrated a variety of behaviours (figure 2), with initial responses varying greatly among the treatments.

### (a) Erratic flight

Erratic behaviour occurred most often under cool phosphor-coated LEDs, although this effect was not significant. There was no significant interaction between light CCT and emission spectra (*n* = 386; *X*^2^ = 0.873, *p* = 0.350), and neither CCT (*X*^2^ = 0.874; *p* = 0.350) nor emission spectra (*X*^2^ = 0.872; *p* = 0.350) were significant predictors of erratic behaviour alone. Temperature and humidity were predictors of erratic flight (temperature: *X*^2^ = 5.010, *p* = 0.025; humidity: *X*^2^ = 5.360, *p* = 0.021); there was a non-significant weak negative correlation between erratic flight and temperature (*t* = −1.014, d.f. = 383, *p* = 0.311, *r* = −0.052) and a non-significant weak positive correlation between erratic flight and humidity (*t* = 1.248, d.f. = 383, *p* = 0.195, *r* = 0.070).

### (b) Phototaxis (flight-to-light)

CCT was a significant predictor of phototaxis, with flight-to-light behaviour more frequent under cool LEDs of either spectrum (*X*^2^ = 9.617, *p* = 0.002). There was no significant interaction between light spectrum and CCT on phototaxis (*X*^2^ = 2.497, *p* = 0.114), and spectrum was not a significant predictor of phototaxis alone (*X*^2^ = 2.644, *p* = 0.104). Temperature and humidity also interacted as significant predictors of phototaxis (*X*^2^ = 5.501, *p* = 0.019).

### (c) Erratic flight and phototaxis (flight-to-light)

When erratic flight and flight-to-light responses were combined, these potentially harmful responses were most pronounced under cool phosphor-coated LEDs (figure 2), with a significant interaction between light CCT and the spectrum (*n* = 386; *X*^2^ = 7.567, *p* = 0.006). Temperature and humidity interacted as near-significant predictors of harmful response behaviour (*X*^2^ = 2.790, *p* = 0.095; individually temperature *X*^2^ = 5.595, *p* = 0.018; humidity *X*^2^ = 4.913, *p* = 0.027); there was a weak non-significant positive correlation between temperature and response (*t* = 1.214, d.f. = 383, *p* = 0.226, *r* = 0.062) and a non-significant weak negative correlation between humidity and response (*t* = −0.946, d.f. = 383, *p* = 0.345, *r* = −0.050). Moon phase was not a significant predictor of moth behaviour in any of the above analyses (erratic flight: *X*^2^ = 5.589, d.f. = 7, *p* = 0.589; phototaxis: *X*^2^ = 9.983, d.f. = 7, *p* = 0.189; unnatural behaviours: *X*^2^ = 9.154, d.f. = 7, *p* = 0.242).

### (d) Geotaxis and latency

Negative geotaxis (upward flight) was more frequent under warm lights than cool lights, although neither CCT nor spectrum were significant predictors (*n* = 199; light spectrum: *X*^2^ = 1.216, *p* = 0.270; CCT: *X*^2^ = 0.369, *p* = 0.544).

On average, latency to respond was 21.45 s (±26.50 s) across treatments. While moths responded the fastest under cool phosphor-coated LEDs (on average 12 s faster than under other treatments, figure 2*b*), the interaction between light CCT and emission spectrum was only a near-significant predictor of latency (*F*_1,326_ = 3.68, *p* = 0.06), but, in isolation, both were highly significant predictors (CCT: *F*_2,326_ = 6.65, *p* = 0.002; spectrum: *F*_1,327_ = 9.13, *p* = 0.003).

### (e) Taxon-specific effects

When all the above analyses were repeated independently with only the four main family groups, the results were qualitatively the same (see electronic supplementary material, figures S2 and S3).

## 4. Discussion

Here we show that a single pulse of ALAN (pALAN) causes substantial shifts in nocturnal moth behavioural responses that are broadly similar across the 64 species tested. When moths were released under control (dark) conditions, 86% flew directly upwards (and likely away from danger); however, following a pulse of light, their behavioural responses were far more varied (just 37%, on average, flew upwards). Moreover, behaviours most likely to be detrimental to moth survival (flight-to-light or erratic flight) were significantly more pronounced, and occurred faster under cool phosphor-coated LEDs, revealing an interaction with pALAN spectrum and correlated colour temperature (CCT).

Many behavioural responses of insects to artificial light sources have been documented anecdotally and experimentally (for a review, see [48]). Most recently, reconstructions of flight kinematics have shown that insects orient their dorsum to the light, which, under unnatural conditions, results in continuous flight towards and around the light [28]. Our finding that cool phosphor-coated LEDs result in erratic flight largely supports this positive phototaxis hypothesis. Upon flight, moths could orient their dorsum to the light, causing blinding and, subsequently, the loss of visual flight stabilization and, thus, uncontrolled erratic flight. However, we note that in numerous cases, erratic behaviour was near-instant, without initial flight towards the source, which may not support this hypothesis. While erratic flight behaviour in isolation was not affected by light treatment, flight-to-light, and the combination of erratic flight and flight-to-light revealed statistically significant trends with pronounced effect sizes. These likely harmful behavioural responses were 1.8 times more likely under cool phosphor-coated LEDs than other lighting treatments, and 25.5 times more likely than under dark control conditions.

Our behavioural assessments cannot determine the underlying mechanisms causing these likely harmful responses, whether due to photobleaching, neurophysiological limitations or maladaptive co-opted behavioural responses (such as flight orientation). Existing knowledge surrounding these mechanisms has typically used static ALAN sources, thus highlighting how future research would benefit from combining the recent advances in three-dimensional kinematic tracking (as seen in [28]) and neural processing [49] to establish whether temporary blindness can explain erratic flight behaviours under pALAN. Studies of neural processing would also allow exploration of the discrepancies in response among light treatments.

We found no evidence for taxonomic differences in moth behavioural responses, contrary to other studies that show family-level biases in attraction to static light sources [50]. Cool phosphor-coated LEDs were consistently the most disruptive, irrespective of family, and this lack of phylogenetic signal suggests that our findings may apply to a wider range of species than the 64 tested here. Further, the diverse nature of behaviours displayed in our study suggests that the typical assessment of the ecological impacts of light, which only considers attraction to light (flight-to-light behaviour/positive phototaxis) [51–53], is limiting. Our results allude to the diversity of responses to pALAN and highlight the importance of considering multiple hypotheses when explaining ecological impacts through behavioural proxies. Extending understanding of behavioural disruption is critical, as these responses are likely associated with changes to ecosystem functioning [11,54], with important ecological impacts.

Further research would benefit from scaling up the monitoring to road verges that experience pALAN and determining the effects on varying life stages (such as caterpillars) in isolation from other sources of artificial light, including streetlights (such as in [12]), and whether critical ecosystem services such as pollination are affected [54]. The nature of vehicle headlights as horizontally focused beams means they create linearly polarized reflections from the flat road surface (particularly when wet [55]). Moths are able to see polarized light [56], and it remains to be determined how polarization and pALAN might interact [48,55,56]. Behavioural work would also benefit from more realistic headlight simulations, with moving lights that gradually increase in brightness, combined with modelling the likelihood of moths being impacted by vehicles. Future work should also seek to increase understanding of pALAN in other systems. For example, given the blue peak of phosphor-coated LEDs is associated with decreasing alertness in older drivers [20], we can speculate that changing the spectral composition of car headlights by reducing the blue peak will likely be associated with beneficial changes to road safety for moths and humans alike.

Our study highlights that pALAN is an emerging form of sensory pollution that may impact moth biodiversity and pollination services across large parts of the landscape that were previously thought to suffer little from the effects of ALAN. These findings, together with evidence from human pupillary reflex and human alertness [21], suggest that it would be beneficial to avoid continued use of cool phosphor-coated LEDs in car headlights.

## Data Availability

Data and R script are included as part of the electronic supplementary material [57].

## References

[1] Falchi F, Cinzano P, Duriscoe D, Kyba CCM, Elvidge CD, Baugh K, Portnov BA, Rybnikova NA, Furgoni R. 2016 The new world atlas of artificial night sky brightness. Sci. Adv. **2**, e1600377. (10.1126/sciadv.1600377) 27386582 PMC4928945

[2] Gaston KJ, Ackermann S, Bennie J, Cox DTC, Phillips BB, Sánchez de Miguel A, Sanders D. 2021 Pervasiveness of biological impacts of artificial light at night. Integr. Comp. Biol. **61**, 1098–1110. (10.1093/icb/icab145) 34169964 PMC8490694

[3] Gaston KJ, Duffy JP, Gaston S, Bennie J, Davies TW. 2014 Human alteration of natural light cycles: causes and ecological consequences. Oecologia **176**, 917–931. (10.1007/s00442-014-3088-2) 25239105 PMC4226844

[4] Gaston KJ, Bennie J, Davies TW, Hopkins J. 2013 The ecological impacts of nighttime light pollution: a mechanistic appraisal. Biol. Rev. **88**, 912–927. (10.1111/brv.12036) 23565807

[5] Wong MKL, Didham RK. 2024 Global meta-analysis reveals overall higher nocturnal than diurnal activity in insect communities. Nat. Commun. **15**, 3236. (10.1038/s41467-024-47645-2) 38622174 PMC11018786

[6] Bennie J, Davies TW, Cruse D, Gaston KJ. 2016 Ecological effects of artificial light at night on wild plants. J. Ecol. **104**, 611–620. (10.1111/1365-2745.12551)

[7] Sanders D, Frago E, Kehoe R, Patterson C, Gaston KJ. 2021 A meta-analysis of biological impacts of artificial light at night. Nat. Ecol. Evol. **5**, 74–81. (10.1038/s41559-020-01322-x) 33139919

[8] Becker A, Whitfield AK, Cowley PD, Järnegren J, Næsje TF. 2013 Potential effects of artificial light associated with anthropogenic infrastructure on the abundance and foraging behaviour of estuary‐associated fishes. J. Appl. Ecol. **50**, 43–50. (10.1111/1365-2664.12024)

[9] de Jong M, Jeninga L, Ouyang JQ, van Oers K, Spoelstra K, Visser ME. 2016 Dose-dependent responses of avian daily rhythms to artificial light at night. Physiol. Behav. **155**, 172–179. (10.1016/j.physbeh.2015.12.012) 26703233

[10] de Jong M, Ouyang JQ, van Grunsven RHA, Visser ME, Spoelstra K. 2016 Do wild great tits avoid exposure to light at night? PLoS One **11**, e0157357. (10.1371/journal.pone.0157357) 27355354 PMC4927185

[11] MacGregor CJ, Pocock MJO, Fox R, Evans DM. 2015 Pollination by nocturnal Lepidoptera, and the effects of light pollution: a review. Ecol. Entomol. **40**, 187–198. (10.1111/een.12174) 25914438 PMC4405039

[12] Boyes DH, Evans DM, Fox R, Parsons MS, Pocock MJO. 2021 Street lighting has detrimental impacts on local insect populations. Sci. Adv. **7**, eabi8322. (10.1126/sciadv.abi8322) 34433571 PMC8386932

[13] Davies TW, Bennie J, Gaston KJ. 2012 Street lighting changes the composition of invertebrate communities. Biol. Lett. **8**, 764–767. (10.1098/rsbl.2012.0216) 22628095 PMC3440964

[14] Spoelstra K, van Grunsven RHA, Donners M, Gienapp P, Huigens ME, Slaterus R, Berendse F, Visser ME, Veenendaal E. 2015 Experimental illumination of natural habitat—an experimental set-up to assess the direct and indirect ecological consequences of artificial light of different spectral composition. Phil. Trans. R. Soc. B **370**, 20140129. (10.1098/rstb.2014.0129) 25780241 PMC4375369

[15] Gaston KJ, Gaston S, Bennie J, Hopkins J. 2015 Benefits and costs of artificial nighttime lighting of the environment. Environ. Rev. **23**, 14–23. (10.1139/er-2014-0041)

[16] Gaston KJ, Holt LA. 2018 Nature, extent and ecological implications of night-time light from road vehicles. J. Appl. Ecol. **55**, 2296–2307. (10.1111/1365-2664.13157) 30147142 PMC6099288

[17] Phillips BB, Bullock JM, Osborne JL, Gaston KJ. 2021 Spatial extent of road pollution: a national analysis. Sci. Total Environ. **773**, 145589. (10.1016/j.scitotenv.2021.145589) 33940735

[18] Grozdanic S, Betts DM, Allbaugh RA, Sakaguchi DS, Kwon YH, Kardon RH, Sonea IM. 2003 Characterization of the pupil light reflex, electroretinogram and tonometric parameters in healthy mouse eyes. Curr. Eye Res. **26**, 371–378. (10.1076/ceyr.26.5.371.15439) 12868018

[19] Loewenfeld IE, Lowenstein O. 1993 The pupil: anatomy, physiology, and clinical applications, 1st edn. Detroit, MI: Wayne State University Press.

[20] Zauner J, Plischke H, Strasburger H. 2022 Spectral dependency of the human pupillary light reflex. Influences of pre-adaptation and chronotype. PLoS One **17**, e0253030. (10.1371/journal.pone.0253030) 35020744 PMC8754338

[21] Tosini G, Ferguson I, Tsubota K. 2016 Effects of blue light on the circadian system and eye physiology. Mol. Vis. **22**, 61–72. http://www.molvis.org/molvis/v22/61 26900325 PMC4734149

[22] Hamdorf K, Höglund G. 1981 Light induced retinal screening pigment migration independent of visual cell activity. J. Comp. Physiol. **143**, 305–309. (10.1007/BF00611166)

[23] Ro AI, Nilsson DE. 1994 Circadian and light-dependent control of the pupil mechanism in tipulid flies. J. Insect Physiol. **40**, 883–891. (10.1016/0022-1910(94)90022-1)

[24] White RH, Banister MJ, Bennett RR. 1983 Spectral sensitivity of screening pigment migration in the compound eye of Manduca sexta. J. Comp. Physiol. **153**, 59–66. (10.1007/BF00610343)

[25] Nordtug T. 1990 Dynamics and sensitivity of the pupillary system in the eyes of noctuid moths. J. Insect Physiol. **36**, 893–901. (10.1016/0022-1910(90)90076-R)

[26] Rukmini AV, Milea D, Baskaran M, How AC, Perera SA, Aung T, Gooley JJ. 2015 Pupillary responses to high-irradiance blue light correlate with glaucoma severity. Ophthalmology **122**, 1777–1785. (10.1016/j.ophtha.2015.06.002) 26299721

[27] Burkhardt DA. 1994 Light adaptation and photopigment bleaching in cone photoreceptors in situ in the retina of the turtle. J. Neurosci. **14**, 1091–1105. (10.1523/JNEUROSCI.14-03-01091.1994) 8120614 PMC6577543

[28] Fabian ST, Sondhi Y, Allen PE, Theobald JC, Lin HT. 2024 Why flying insects gather at artificial light. Nat. Commun. **15**, 689. (10.1038/s41467-024-44785-3) 38291028 PMC10827719

[29] Walcott B. 1969 Movement of retinula cells in insect eyes on light adaptation. Nature **223**, 971–972. (10.1038/223971a0) 5803405

[30] Meyer-Rochow VB. 1974 Fine structural changes in dark-light adaptation in relation to unit studies of an insect compound eye with a crustacean-like rhabdom. J. Insect Physiol. **20**, 573–589. (10.1016/0022-1910(74)90164-4) 4819574

[31] Shimoda M, Honda K ichiro. 2013 Insect reactions to light and its applications to pest management. Appl. Entomol. Zool. **48**, 413–421. (10.1007/s13355-013-0219-x)

[32] Yabu T, Miyashita N, Uematsu S, Wakakuwa M, Arikawa K. 2014 Suppression of activity and compound eye spectral sensitivity of two noctuid moths, Helicoverpa armigera and Mamestra brassicae under flickering green light. Jpn. J. Appl. Entomol. Zool. **58**, 211–216. (10.1303/jjaez.2014.211)

[33] Stockman A, Sharpe LT. 2000 The spectral sensitivities of the middle- and long-wavelength-sensitive cones derived from measurements in observers of known genotype. Vis. Res. **40**, 1711–1737. (10.1016/s0042-6989(00)00021-3) 10814758

[34] Troscianko J, Stevens M. 2015 Image calibration and analysis toolbox—a free software suite for objectively measuring reflectance, colour and pattern. Meth. Ecol. Evol. **6**, 1320–1331. (10.1111/2041-210X.12439) PMC4791150 27076902

[35] Briolat ES, Gaston KJ, Bennie J, Rosenfeld EJ, Troscianko J. 2021 Artificial nighttime lighting impacts visual ecology links between flowers, pollinators and predators. Nat. Commun. **12**, 4163. (10.1038/s41467-021-24394-0) 34230463 PMC8260664

[36] Johnsen S, Kelber A, Warrant E, Sweeney AM, Widder EA, Lee RL Jr, Hernández-Andrés J. 2006 Crepuscular and nocturnal illumination and its effects on color perception by the nocturnal hawkmoth Deilephila elpenor. J. Exp. Biol. **209**, 789–800. (10.1242/jeb.02053) 16481568

[37] Eguchi E, Watanabe K, Hariyama T, Yamamoto K. 1982 A comparison of electrophysiologically determined spectral responses in 35 species of Lepidoptera. J. Insect Physiol. **28**, 675–682. (10.1016/0022-1910(82)90145-7)

[38] Macgregor CJ, Evans DM, Fox R, Pocock MJO. 2017 The dark side of street lighting: impacts on moths and evidence for the disruption of nocturnal pollen transport. Glob. Chang. Biol. **23**, 697–707. (10.1111/gcb.13371) 27251575

[39] Waring P, Townsend M, Lewington R. 2018 Field guide to the moths of Great Britain and Ireland. London, UK: Bloomsbury Publishing.

[40] Sterling P, Parsons M, Lewington R. 2023 Field guide to the micro-moths of Great Britain and Ireland, 2nd edn. London, UK: Bloomsbury Publishing.

[41] R Core Team. 2023 R: a language and environment for statistical computing. R Foundation for Statistical Computing. Vienna, Austria. See https://www.R-project.org/.

[42] Wickham H *et al*. 2019 Welcome to the {tidyverse}. J.O.S.S. **4**, 1686. (10.21105/joss.01686)

[43] Maia R, Gruson H, Endler JA, White TE. 2019 PAVO 2: new tools for the spectral and spatial analysis of colour in R. Methods Ecol. Evol. **10**, 1097–1107. (10.1111/2041-210X.13174)

[44] Ripley BD. 2002 Modern applied statistics with S. New York, NY: Springer.

[45] Pinheiro J. 2011 Nlme: linear and nonlinear mixed effects models. R package version 3. See https://CRAN.R-project.org/package=nlme.

[46] Bates D, Machler M, Bolker B, Walker S. 2015 Fitting linear mixed-effects models using {lme4} journal of statistical software. J. Stat. Softw. **67**, 1–48. (10.18637/jss.v067.i01)

[47] Kolligs D. 2000 Okologische Auswirkungen kunstlicher Lichtquellen auf nachtaktive Insekten, insbesondere Schmetterlinge (Lepidoptera). See https://www.researchgate.net/publication/292713590_Okologische_Auswirkungen_kunstlicher_Lichtquellen_auf_nachtaktive_Insekten_insbesondere_Schmetterlinge_Lepidoptera

[48] Boyes DH, Evans DM, Fox R, Parsons MS, Pocock MJO. 2021 Is light pollution driving moth population declines? A review of causal mechanisms across the life cycle. Insect Conserv. Divers. **14**, 167–187. (10.1111/icad.12447)

[49] Stöckl AL, O’Carroll D, Warrant EJ. 2017 Higher-order neural processing tunes motion neurons to visual ecology in three species of hawkmoths. Proc. R. Soc. B **284**, 20170880. (10.1098/rspb.2017.0880) PMC5489734 28637860

[50] Somers-Yeates R, Hodgson D, McGregor PK, Spalding A, Ffrench-Constant RH. 2013 Shedding light on moths: shorter wavelengths attract noctuids more than geometrids. Biol. Lett. **9**, 20130376. (10.1098/rsbl.2013.0376) 23720524 PMC3730649

[51] Pawson SM, Bader MKF. 2014 LED lighting increases the ecological impact of light pollution irrespective of color temperature. Ecol. Appl. **24**, 1561–1568. (10.1890/14-0468.1) 29210222

[52] van Grunsven RHA, Becker J, Peter S, Heller S, Hölker F. 2019 Long-term comparison of attraction of flying insects to streetlights after the transition from traditional light sources to light-emitting diodes in urban and peri-urban settings. Sustainability **11**, 6198. (10.3390/su11226198)

[53] Wakefield A, Broyles M, Stone EL, Harris S, Jones G. 2018 Quantifying the attractiveness of broad‐spectrum street lights to aerial nocturnal insects. J. Appl. Ecol. **55**, 714–722. (10.1111/1365-2664.13004)

[54] Macgregor CJ, Scott-Brown AS. 2020 Nocturnal pollination: an overlooked ecosystem service vulnerable to environmental change. Emerg. Top. Life Sci. **4**, 19–32. (10.1042/ETLS20190134) 32478390 PMC7326339

[55] Horváth G, Kriska G, Malik P, Robertson B. 2009 Polarized light pollution: a new kind of ecological photopollution. Front. Ecol. Environ. **7**, 317–325. (10.1890/080129)

[56] Belušič G, Šporar K, Meglič A. 2017 Extreme polarisation sensitivity in the retina of the corn borer moth Ostrinia. J. Exp. Biol. **220**, 2047–2056. (10.1242/jeb.153718) 28341662

[57] Fabusova M, Gaston K, Troscianko J. 2024 Data from: pulsed artificial light at night alters moth flight behaviour. Figshare. (10.6084/m9.figshare.c.7524698) PMC11557245 39532146

